# Preparing High-Strength Formed Coke from Coke Breeze Using a Composite Binder: Complementary Integration of Phenolic Resin Binding and Coal Tar Pitch Carbonization

**DOI:** 10.3390/ma19183843

**Published:** 2026-09-09

**Authors:** Lin Wang, Yongbin Yang, Yinrui Dong, Yan Zhang, Shichao He, Qian Li

**Affiliations:** School of Minerals Processing and Bioengineering, Central South University, Changsha 410083, China; wanglin@csu.edu.cn (L.W.);

**Keywords:** composite binder, coke, fossil energy, carbonization, solid waste, coal tar pitch

## Abstract

Coke breeze, a solid waste generated during coke production, transportation and utilization, possesses physicochemical properties akin to metallurgical coke except for its finer particle size. Recycling this material offers an effective route to conserve and reuse coal resources, yielding notable environmental and economic benefits. In this study, a composite binder composed of phenolic resin (PR) and coal tar pitch (CTP) was developed for producing high-strength metallurgical coke substitutes from coke breeze. This strategy complementarily combines the excellent binding and curing properties of PR with the superior high-temperature carbonization performance of CTP. Thermal and structural analyses elucidated distinct pyrolysis characteristics and functional group distributions between the two binders, providing a theoretical foundation for their complementary interaction. Systematic investigations into briquette strength, performance, and microstructure established that the optimal composite binder dosage is 15 wt.% of coke breeze, with a CTP-to-PR mass ratio of 7:3. This complementary system substantially enhanced the compressive strength of the formed coke, reaching 37.73 MPa after carbonization at 1000 °C, and improved its thermal stability, as reflected by a Coke Strength after Reaction (CSR) of 41.21%. Compared to the individual binders, the composite binder exhibited superior consolidation efficiency and structural integrity, presenting a promising approach for solid waste valorization and sustainable coke production.

## 1. Introduction

Coal, as a fossil fuel, remains the dominant energy source globally, playing a critical role in power generation and metallurgical industries [1,2,3]. However, the depletion of high-quality coal reserves and subsequent price surges have underscored the necessity of sustainable resource utilization, including recycling coal-derived byproducts [4,5]. Coke breeze, a fine particulate waste generated during metallurgical coke production (coal’s highest value-added derivative) [6,7], exhibits physicochemical properties comparable to commercial coke except for its smaller particle size [8,9,10]. Its suboptimal combustion performance, characterized by low efficiency due to flue gas entrainment and potential equipment abrasion, limits direct utilization [11]. Agglomerating coke breeze into formed coke presents a viable strategy for both waste valorization and partial substitution of premium metallurgical coke, thereby addressing resource conservation imperatives. Compared with pulverized coal, coke breeze has high fixed carbon and low volatile content [12]. The strengths of briquettes using pulverized coal do not meet the requirements of metallurgical quality coke. In addition, their high carbonization temperatures and significant weight losses (30–60%) are uneconomical [13,14,15]. Therefore, coke breeze is more suitable as the raw material to prepare briquettes for reducing the consumption of metallurgical coke.

Phenolic resin (PR) is widely recognized as an effective binder for various materials due to its superior binding properties [16,17,18,19]. However, despite enabling coke breeze briquettes to achieve high compressive strength (e.g., 70 MPa), its high-temperature carbonization behavior remains poorly understood, and its industrial application is constrained by high costs [20,21,22,23,24,25]. While compressive strength is a commonly reported metric, it is a nonstandard parameter for evaluating coke quality, as it varies significantly with sample dimensions, shape, and testing apparatus. Consequently, it may serve as a preliminary screening criterion but lacks reliability for definitive performance assessment. In contrast, standardized coke quality indices, such as the Coke Reactivity Index (CRI) and Coke Strength after Reaction (CSR), are indispensable for evaluating functional performance under industrial conditions. Future research should prioritize these industry-relevant parameters to ensure accurate and practical binder assessment, bridging the gap between laboratory findings and real-world applicability.

In contrast to PR, coal tar pitch (CTP) is a cost-effective binder widely employed in graphite electrodes, carbon fiber, and activated carbon production [26,27,28,29,30,31,32]. As a byproduct of the coal-coking process, CTP presents a theoretically viable alternative for producing metallurgical-grade briquettes [33]. However, its lack of adhesion at room temperature limits its direct application in coke breeze briquetting [34,35], necessitating modifications to enhance its binding properties under ambient conditions. Previous studies have demonstrated that additives such as trimethylbenzene can improve CTP’s room-temperature adhesion [6,36,37], yet the resulting briquettes remain unsuitable for fully replacing metallurgical coke in blast furnaces or cupolas. To bridge this gap, further optimization is required to enhance both the mechanical and thermal strength of coke breeze-based formed coke.

Based on the above analysis, PR exhibits excellent adhesive properties, but its high-temperature carbonization performance remains unclear, and its dosage is excessive when used for producing formed coke. In contrast, coal tar pitch demonstrates relatively weak adhesion at room temperature but superior carbonization behavior at elevated temperatures. Therefore, this study proposes an approach employing low-dose PR as a binder combined with coal tar pitch as a high-temperature carbonizing agent. This synergistic system establishes bridging connections between coke breeze particles within the formed coke, leveraging the adhesive capability of PR while developing the carbon skeleton formed through pitch carbonization. Such a dual-phase bonding mechanism is designed to enhance the carbonization and consolidation of formed coke. Experiments were carried out for preparing formed coke products using coke breeze with coal tar pitch alone, PR alone and their mixtures as the binder. The performance of the obtained products was carefully tested and compared. A composite binder was developed for producing a product with higher strength than coal tar pitch and PR alone as the binder.

While the combination of coal tar pitch and phenolic resin for coke breeze briquetting has been explored in earlier foundational studies [22,23], these works primarily focused on tensile strength measurements of laboratory-scale briquettes and employed relatively high binder dosages (20–35 wt.%) with PR as the dominant component. Moreover, a systematic optimization of the devolatilization step, evaluation of high-temperature thermal performance using industry-standard CRI/CSR metrics, and in-depth mechanistic characterization of the binder-derived carbon structure remain unexplored. To bridge this gap, the present study systematically investigates an optimized CTP-dominant composite binder system with lower total dosage, evaluates the resulting formed coke using both compressive strength and standardized CRI/CSR tests, and comprehensively elucidates the synergistic strengthening mechanism through combined SEM, Raman, and XRD analyses. This integrated approach provides both practical and scientific advancements over previous reports.

## 2. Materials and Methods

### 2.1. Materials

The coke breeze, selected from a local steel industry in Nanjing of Jiangsu province (China), was used as the raw material in the tests. Table 1 shows the proximate analysis and particle size composition of coke breeze. It can be seen that the particle size of the coke breeze was too small, which was not conducive to the production of the briquettes [6]. The strength of the prepared briquette can be improved effectively by appropriately increasing the proportion of large particle coke breeze (0.5~3 mm). It was worth pointing out that the coke breeze used for each experiment was re-portioned according to Table 1 to stabilize the particle size distribution of the samples.

The coal tar pitch (CTP) selected from Nanjing of Jiangsu province (China) was used as all tests’ main binder. Table 2 shows the proximate analysis, ultimate analysis and softening temperature of CTP and phenolic resin (PR, from Shanghai of China). CTP was crushed into fine particles less than 0.15 mm in size so that it could be better mixed with coke breeze and PR powder. Meanwhile, a trimethylbenzene reagent with 99% purity bought from Shanghai Macklin Biochemical Co., Ltd. (China) was used as an activator to improve the low-temperature binding of CTP. PR powder used in this study as the auxiliary binder was from Henan province. It was worth pointing out that it was an inexpensive PR powder with poorer binding than the liquid PR [20].

### 2.2. Experimental Method

**Figure 1:**
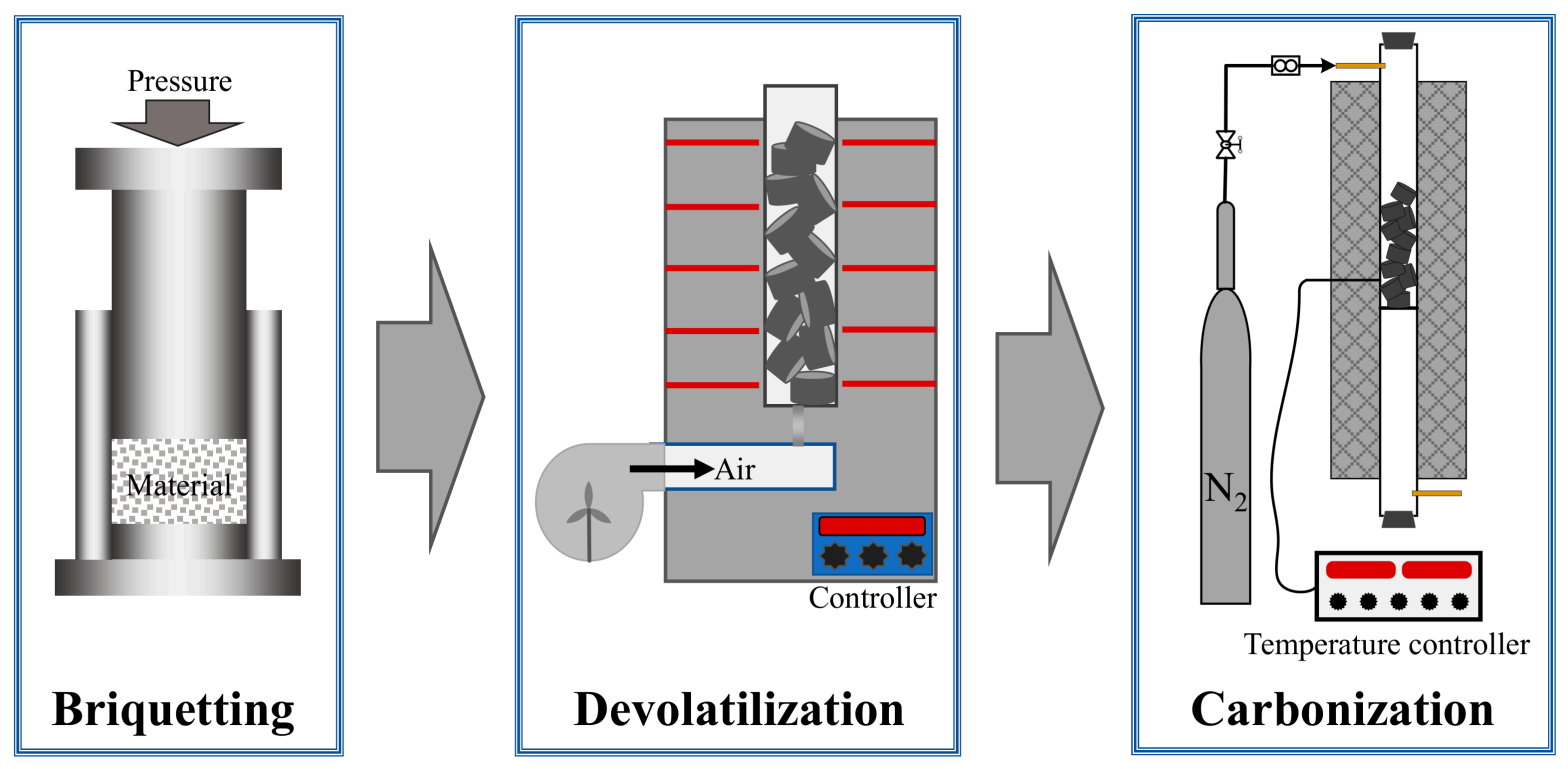
Schematic diagram of test process and equipment.

The schematic diagram of the experiment process and equipment is illustrated in Figure 1. The coke breeze, CTP and PR powder were first mixed carefully in the appropriate proportions. Then, 7 g of the mixture (with 0.2%wt trimethylbenzene and 10%wt water) was briquetted into a cylindrical green briquette with a bottom diameter of 20 mm by the appropriate pressures. The devolatilization and carbonization processes were carried out in a ventilated and vertical furnace (GSL-800X-RF, Manufactured by Hefei Kejing Materials Technology Co., Ltd., Hefei, China). The obtained formed coke products were detected by various tests. Their compressive strengths were tested by a compression testing machine from ‌‌Jinan Times New Light Instrument Co., Ltd in Jinan, China (GB/T 7314-2017 [38]). Their ultimate and proximate analyses were performed by an Elementar-UNICUBE from Hanau, Germany and an Industrial analyzer (SDTGA5000a, ‌from Hunan Sundy Science And Technology Co., Ltd.‌ in Changsha, China), respectively. And their morphology was observed by optical microscopy (Leica DMRXP, from Shimadzu Corporation in Kyoto, Japan), a scanning electron microscope equipped with an energy-dispersive X-ray system (SEM, FEI Quanta 200). A thermogravimetric (TG) with differential scanning calorimetry (DSC) analyzer (NETZSCH STA409C/PC, from the company ‌NETZSCH in Bavaria, Germany.) was used to investigate the thermochemical properties of coal tar pitch (CTP) and phenolic resin powder (PR). A Perkin Elmer Spectrum 100 (from Thermo Fisher Scientific, Inc., based in Waltham, MA, USA) was used to collect the FT-IR spectra of the binders’ structures, and they were collected in the range of 4000–500 cm^−1^ absorption band with 4 cm^−1^ resolution.

### 2.3. Data Analysis

In the experiments, the compressive strength of briquette (pi) was calculated as:

(1) pi = Fi/mi

where Fi is the maximum force the briquette can withstand before breaking, mi is the bottom area of the cylindrical briquette (314 mm^2^). This study evaluated the thermal performance of formed coke products in accordance with the Chinese national standard GB/T 4000-2017 [39]. The CRI was calculated as:

(2) CRI = (g0 − gi)/g0 × 100%

where g0 is the total weight of the tested briquettes, gi is the weight of the briquettes after reaction with CO_2_. The CSR was calculated as:

(3) CSR = g10/gi × 100%

where gi is the weight of the briquettes after reaction with CO_2_, g10 is the weight of particles larger than 10 mm of briquettes after reaction with CO_2_ and rotating through the drum.

## 3. Results and Discussion

### 3.1. Thermal Behaviors and Structure Information of the Binders

#### 3.1.1. Thermal Behaviors of the Binders

**Figure 2:**
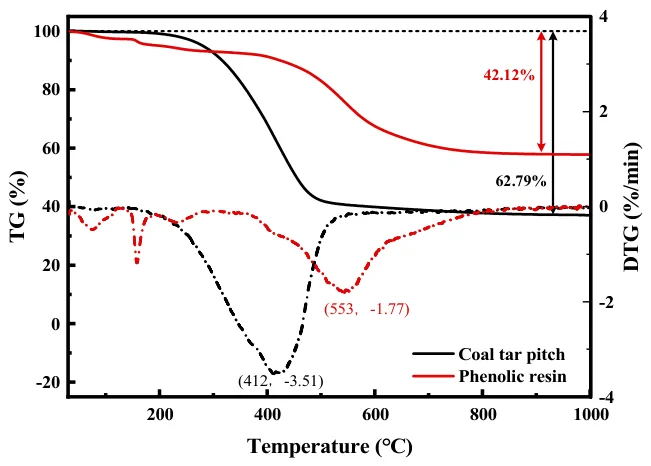
Thermal behaviors of the coal tar pitch and phenolic resin under nitrogen.

Figure 2 shows the thermal behaviors of the two binders under nitrogen. The total mass loss ratios of CTP and phenolic resin were 62.79% and 41.34%, respectively, which are close to their volatile matter contents (60.02% and 41.21%). Meanwhile, the mass loss rates were more clearly reflected in the DTG curves. CTP exhibited a single-peak mass loss characteristic, whereas phenolic resin showed three distinct peaks. The maximum mass loss rate of CTP reached -3.51%/min at 410 °C, while all peak values of phenolic resin were lower than this, indicating a more intense pyrolysis reaction in CTP. Furthermore, the thermal decomposition of CTP occurred mainly in the range of 200–550 °C, with significant mass loss due to its high volatile content. Above 550 °C, the mass loss stabilized, indicating the gradual formation of a stable carbon structure. In contrast, phenolic resin showed slight mass loss between room temperature and 200 °C, primarily attributed to the removal of adsorbed moisture. Between 200 °C and 600 °C, significant decomposition occurred accompanied by an exothermic peak, reflecting the formation of a cross-linked structure and further pyrolysis. Above 600 °C, the material stabilized, with the residue consisting mainly of a carbon skeleton. The thermal behaviors of the two binders differed significantly. Compared to CTP, which undergoes a more violent devolatilization process, phenolic resin as a binder may cause less damage to the internal structure of the briquette during devolatilization.

#### 3.1.2. Structure Information Determined by FT-IR

**Figure 3:**
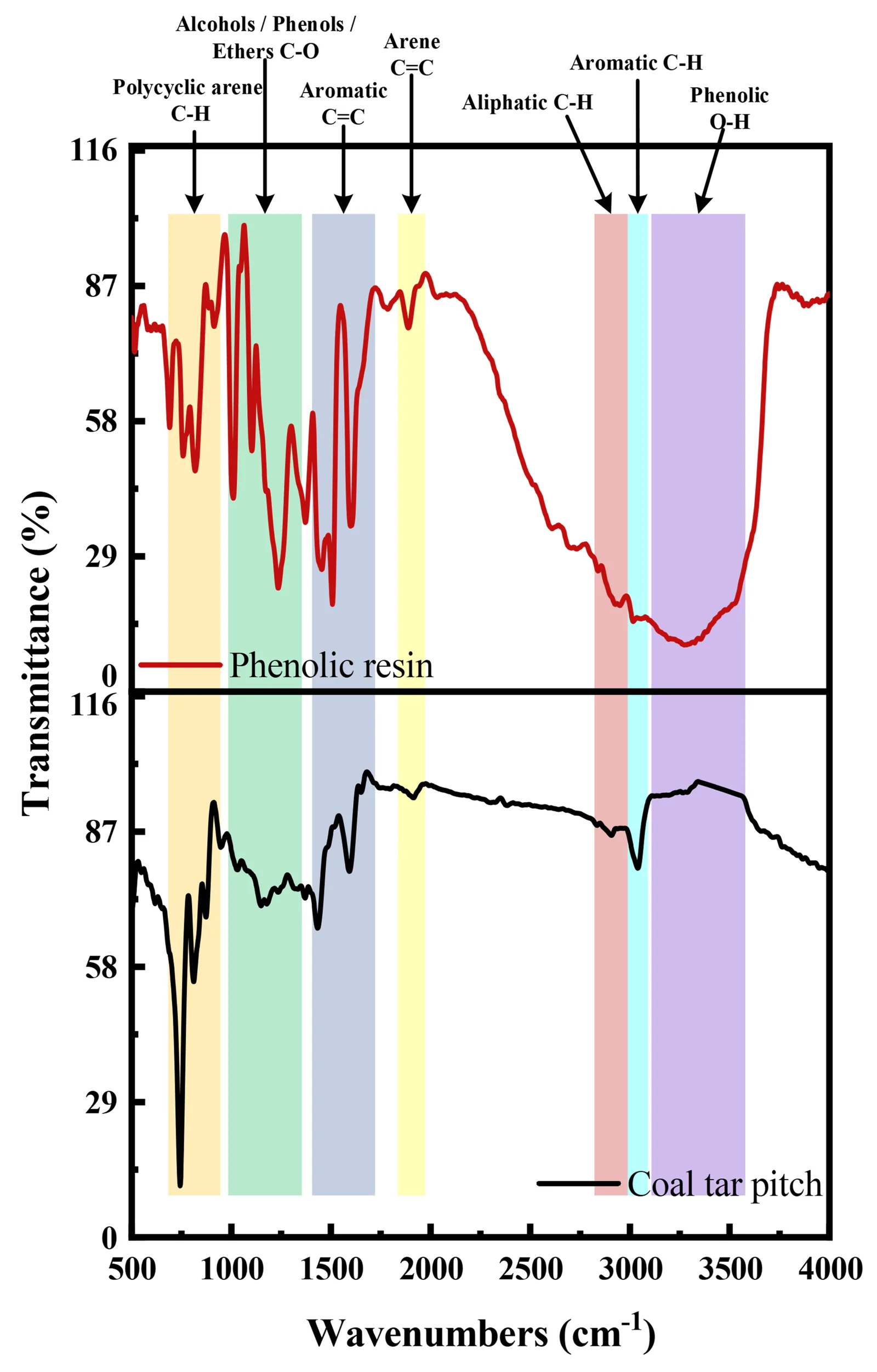
FTIR spectra of the coal tar pitch and phenolic resin.

As shown in Figure 3, the prominent peak at 744 cm^−1^ is primarily attributed to the C-H (polycyclic arene) wagging vibrations abundant in CTP. Additionally, characteristic absorption bands for aliphatic C-H stretching vibrations (2700–3000 cm^−1^), aromatic C-H stretching vibrations (3000–3200 cm^−1^), and hydroxyl O-H stretching vibrations (3200–3700 cm^−1^) were observed in phenolic resin. It is noteworthy that the intensity of the aromatic C=C stretching vibration in phenolic resin was significantly stronger than that in CTP, suggesting its superior binding capability [37,40]. However, the aromatic C-H stretching vibration intensity in phenolic resin was considerably weaker, indicating lower reactivity for further polycondensation, which may result in inferior carbonization and consolidation performance [9,40]. This complementary structural feature underpins the rationale of the present study: by synergistically utilizing PR’s excellent binding and curing properties alongside CTP’s superior high-temperature carbonization and consolidation behavior, it is possible to produce formed coke with substantially enhanced mechanical strength. This strategy integrates the advantages of both binders, thereby providing an effective route for improving the quality of coke breeze-based products.

### 3.2. Consolidation System of Formed Coke Prepared Using Coke Breeze

#### 3.2.1. Consolidation System of Briquette Using Coal Tar Pitch as Binder

##### Effects of Coal Tar Pitch Dosage and Briquetting Pressure

**Figure 4:**
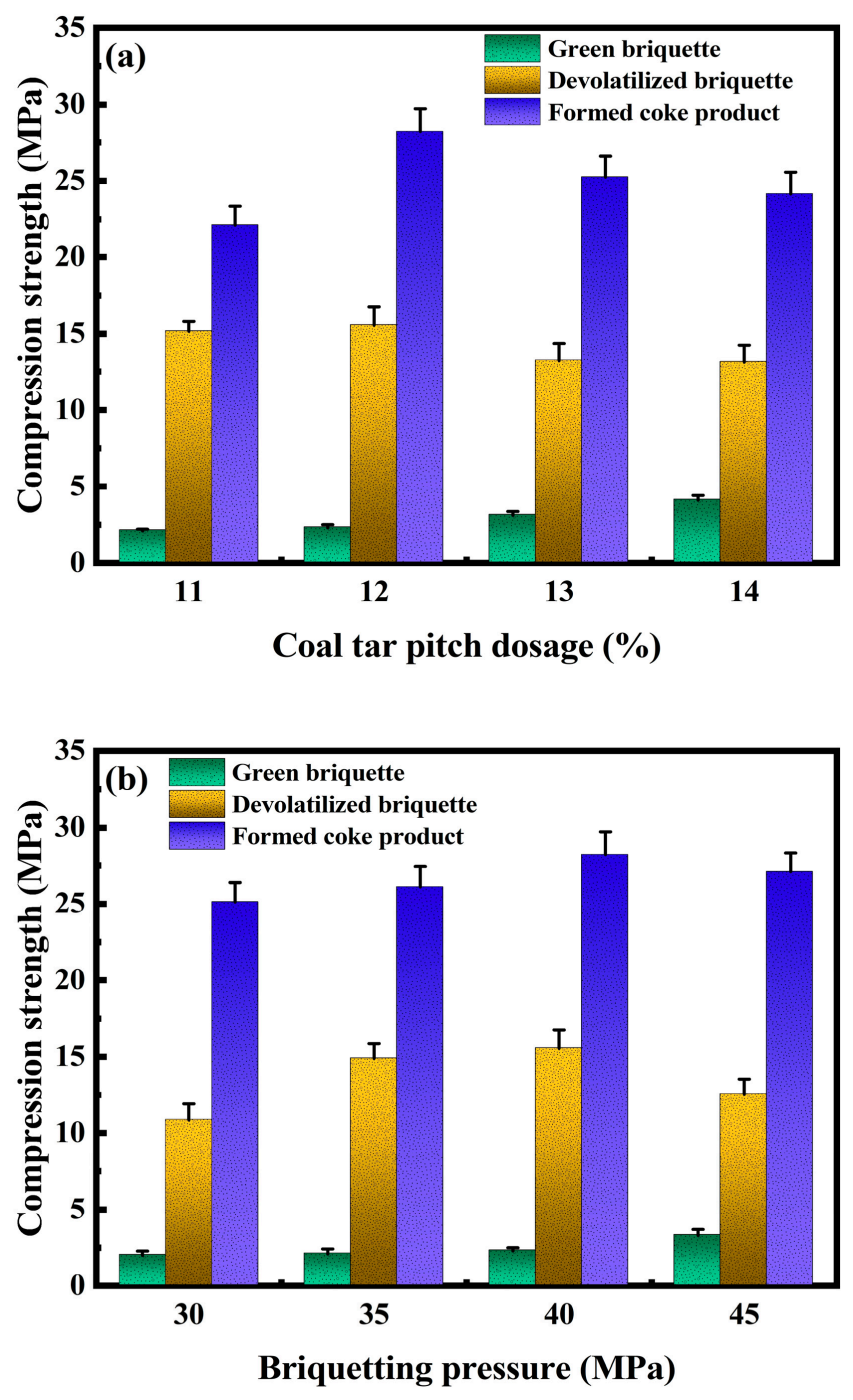
The effects of coal tar pitch dosage (**a**) and briquetting pressure (**b**) on the compressive strengths of briquettes. Devolatilizing condition: T = 370 °C, Time = 25 min. Carbonizing condition: T = 700 °C, Time = 30 min.

The consolidation performance of briquettes using coal tar pitch (CTP) as the binder was first investigated. Due to its poor adhesion at room temperature, CTP was activated with trimethylbenzene to enhance its binding capability during the briquetting process. The effects of CTP dosage (with 40 MPa briquetting pressure) and briquetting pressure (with 12% CTP dosage) on the compressive strengths of briquettes are shown in Figure 4.

The CTP dosage proved to be a critical factor influencing briquette strength. As illustrated in Figure 4a, the compressive strength of the green briquette increased from 2.10 MPa at an 11% CTP dosage to 4.10 MPa at a 14% dosage. This enhancement can be attributed to the greater cohesive force provided by the additional binder, which promoted stronger adhesion between the coke breeze particles. A similar trend was observed for the thermally treated briquettes. The compressive strengths of both the devolatilized and carbonized briquettes increased initially, reaching optimal values of 15.55 MPa and 28.21 MPa, respectively, at a 12% CTP dosage, up from 15.15 MPa and 22.12 MPa at the 11% dosage. This strength development primarily relied on the improved initial strength of the green briquette, which provided a more robust matrix for subsequent thermal processing. However, a further increase in the CTP dosage beyond 12% led to a decline in the strength of the devolatilized and carbonized briquettes. This indicates that the optimal dosage for trimethylbenzene-activated CTP had been exceeded. The excessive binder introduced a higher volume of volatile components, which resulted in a more violent devolatilization reaction. This intense reaction generated more pores within the briquette structure, leading to a looser internal architecture in the devolatilized briquette and consequently reducing its mechanical strength. As the strength of the final formed coke product (carbonized briquette) is heavily dependent on the structural integrity of its devolatilized precursor, their strength trends were closely correlated.

In industrial briquetting operations, forming pressure serves as a critical process parameter that profoundly influences the strength of green briquettes. Figure 4b illustrates the effect of briquetting pressure on the compressive strength of green, devolatilized, and carbonized briquettes. As the pressure increased from 30 MPa to 40 MPa, the compressive strengths rose from 2.00 MPa, 10.84 MPa, and 25.12 MPa to 2.30 MPa, 15.55 MPa, and 28.21 MPa, respectively. This improvement can be attributed to the reduction in porosity under higher pressure, which enhances the briquette density and increases the contact area between coke breeze particles. The compacted structure strengthens particle interlocking and frictional forces, while also improving binder-particle adhesion, thereby contributing to greater mechanical strength. However, a further increase in briquetting pressure beyond 40 MPa resulted in a noticeable decline in the strength of both devolatilized and carbonized briquettes. This reduction is likely due to the excessively high density of the green briquettes formed under such pressure. The highly compacted structure impeded the effective and rapid release of volatiles generated from the CTP binder during the subsequent thermal treatment stages. The trapped gases increased internal stress within the briquettes, leading to microstructural damage and, consequently, a deterioration in mechanical strength.

##### Effects of Devolatilizing and Carbonizing Conditions

**Figure 5:**
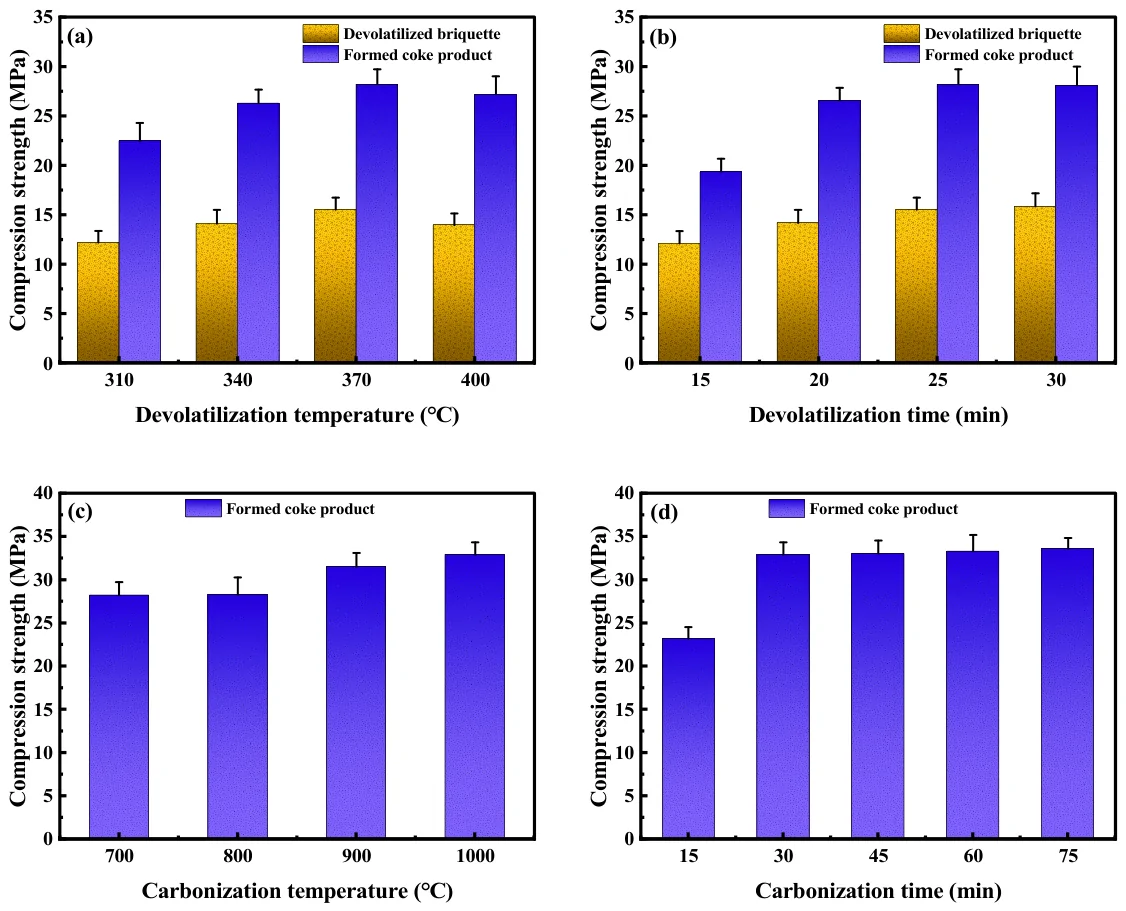
The effects of devolatilization temperature (**a**) and time (**b**), and carbonization temperature (**c**) and time (**d**) on the compressive strengths of briquettes. Coal tar pitch dosage: 12%; Briquetting pressure: 40 MPa.

The effects of devolatilization and carbonization conditions on the mechanical strength of the briquettes were systematically investigated. As shown in Figure 5a,b, both devolatilization temperature and time exerted a significant influence on the compressive strength of the devolatilized briquettes and the final formed coke products. Notably, the strength trends of the formed coke closely mirrored those of the devolatilized briquettes, indicating that the structural integrity established during devolatilization plays a decisive role in the quality of the final product. The compressive strength of both the devolatilized briquettes and the formed coke increased with rising devolatilization temperature, reaching optimum values of 15.55 MPa and 28.21 MPa, respectively, at 370 °C. This improvement can be attributed to enhanced volatilization and pyrolysis of the binder at elevated temperatures, which promotes curing and facilitates the subsequent carbonization process. However, further increasing the devolatilization temperature beyond 370 °C led to a decline in strength. This deterioration is likely due to the overly rapid release of volatiles, which generates excessive internal stresses and causes microstructural damage within the briquettes. The carbonization temperature also demonstrated a profound impact on product strength. As illustrated in Figure 5c, the compressive strength of the formed coke increased steadily with carbonization temperature, attaining a maximum of 32.92 MPa at 1000 °C. This trend confirms that higher temperatures promote more complete carbonization and structural consolidation of the carbon skeleton. Similarly, extending the carbonization time from 15 to 30 min resulted in a significant strength increase from 28.21 MPa to 32.92 MPa, as shown in Figure 5d, underscoring that the carbonization process is time-dependent. However, beyond 30 min, the strength exhibited a negligible change, suggesting that the carbonization reaction had approached completion under the given conditions. Therefore, the optimum devolatilization and carbonization conditions for preparing formed coke products using CTP as the binder were determined to be 370 °C for 25 min and 1000 °C for 30 min, respectively.

#### 3.2.2. Consolidation System of Briquette Using Phenolic Resin as Binder

##### Effects of Phenolic Resin Dosage and Briquetting Pressure

**Figure 6:**
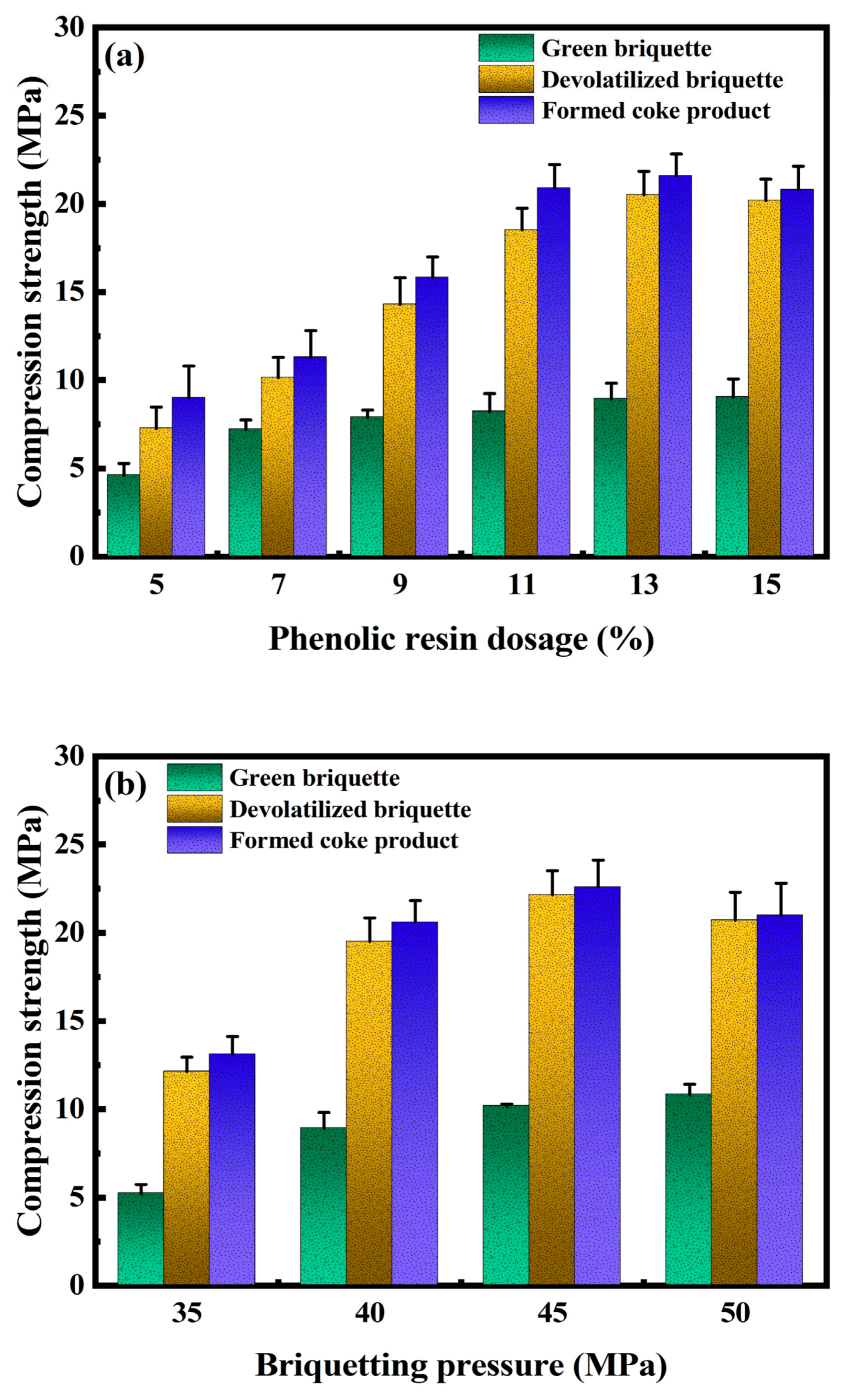
The effects of phenolic resin dosage (**a**) and briquetting pressure (**b**) on the compressive strengths of briquettes. Devolatilizing condition: T = 370 °C, Time = 25 min. Carbonization condition: T = 700 °C, Time = 30 min.

The consolidation behavior of briquettes using phenolic resin (PR) as the sole binder was subsequently evaluated. Figure 6 illustrates the effects of PR dosage (under 40 MPa briquetting pressure) and briquetting pressure (with 13% PR dosage) on the compressive strengths of the briquettes at different processing stages.

As shown in Figure 6a, the PR dosage significantly influenced the briquette strength. The compressive strength of the green briquette increased markedly from 4.59 MPa at a 5% PR dosage to 9.02 MPa at a 15% dosage. This enhancement is attributed to the greater cohesive force provided by the additional resin, which improves the adhesion between coke breeze particles. Notably, the green briquettes prepared with PR exhibited substantially higher strength than those with CTP under identical conditions, confirming the superior binding capability of PR at room temperature. This observation aligns with the FT-IR structural analysis presented in Section 3.1.2, which indicated a stronger aromatic C=C stretching vibration in PR, correlating with its enhanced binding potential. The compressive strengths of the devolatilized and carbonized briquettes also initially increased with PR dosage, reaching maximum values of 20.51 MPa and 21.59 MPa, respectively, at a 13% PR dosage, compared to 7.26 MPa and 8.98 MPa at the 5% dosage. This improvement stems from the stronger initial green strength provided by the PR matrix. However, further increasing the PR dosage beyond 13% led to a decline in strength, establishing 13% as the optimal dosage. It is noteworthy that at the same binder dosage, the devolatilized briquettes prepared with PR exhibited higher strength than those with CTP, underscoring PR’s superior binding and curing characteristics. Nevertheless, the strength gain of the formed coke after carbonization at 700 °C was limited and lower than that achieved with CTP, indicating inferior carbonization and consolidation performance of PR at elevated temperatures. This finding is consistent with the FT-IR analysis in Section 3.1.2, which suggested lower reactivity for polycondensation in PR, likely contributing to its weaker high-temperature structural development.

The effect of briquetting pressure on the strength of briquettes prepared with 13% phenolic resin is shown in Figure 6b. As the pressure increased from 35 MPa to 45 MPa, the compressive strengths of the green, devolatilized, and carbonized briquettes increased significantly, from 5.23 MPa, 12.12 MPa, and 13.09 MPa to 10.18 MPa, 22.15 MPa, and 22.59 MPa, respectively. This trend underscores the critical role of adequate pressure in enhancing particle packing and interlocking, thereby improving the mechanical integrity across all processing stages. However, a further increase in briquetting pressure beyond 45 MPa led to a noticeable decline in the strength of both the devolatilized and carbonized briquettes, establishing 45 MPa as the optimal briquetting pressure for the PR-bound system. This optimum pressure is higher than that determined for CTP-based briquettes. The difference may be attributed to the lower volatile content of phenolic resin, which results in a gentler devolatilization process. This gentler devolatilization process minimizes internal structural damage, allowing briquettes with higher green density (achieved under higher briquetting pressure) to better maintain their structural integrity during subsequent thermal treatment.

##### Effects of Devolatilizing and Carbonizing Conditions

**Figure 7:**
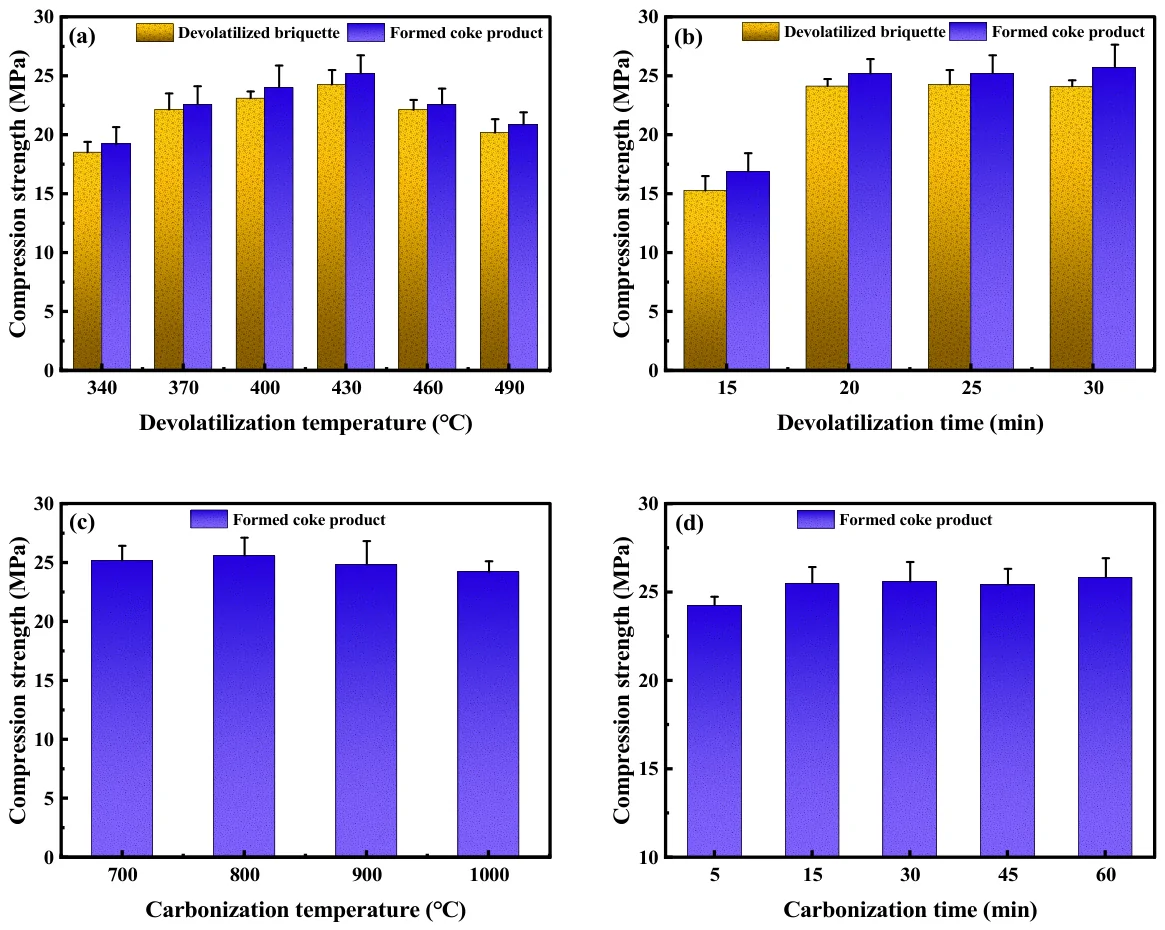
The effects of devolatilization temperature (**a**) and time (**b**), and carbonization temperature (**c**) and time (**d**) on the compressive strengths of briquettes. Phenolic resin dosage: 13%; Briquetting pressure: 45 MPa.

The effects of devolatilization and carbonization conditions on the mechanical strength of the briquettes prepared by PR as the binder using coke breeze were systematically investigated, as shown in Figure 7.

As illustrated in Figure 7a, the compressive strengths of both the devolatilized and carbonized briquettes increased with rising devolatilization temperature, reaching maximum values of 24.26 MPa and 25.22 MPa, respectively, at 430 °C. However, further increasing the temperature beyond this point resulted in a decline in strength, establishing 430 °C as the optimal devolatilization temperature for the PR-bound system. This optimum temperature is higher than that observed for CTP-based briquettes, which can be attributed to the lower volatile content of PR. The milder devolatilization behavior reduces the intensity of internal reactions and structural disruption, thereby permitting the use of higher temperatures to enhance the curing of the PR matrix without compromising briquette integrity. The influence of devolatilization time on compressive strength is presented in Figure 7b. The optimal duration was found to be 20 min, with the devolatilized and carbonized briquettes achieving strengths of 24.13 MPa and 25.19 MPa, respectively—a significant increase from the 15.28 MPa and 16.89 MPa recorded at 15 min.

Notably, after carbonization at 700 °C, the strength difference between the formed coke and the devolatilized briquette was minimal, especially when compared to the pronounced strength gain in CTP-based briquettes. This suggests that higher carbonization temperatures have a limited effect on enhancing the strength of PR-based products. This observation is further supported by the data in Figure 7c,d. As the carbonization temperature increased from 700 °C to 1000 °C, the compressive strength of the formed coke did not improve and in some cases even decreased, confirming the poor carbonization consolidation performance of this PR powder. Consequently, the effective window for carbonization is narrower, leading to a shorter optimal carbonization time of just 15 min.

Based on these findings, it is concluded that for formed coke products using PR as the binder, the binding and curing effects are primarily achieved during the devolatilization stage, with no significant strength enhancement attained through higher carbonization temperatures. The resulting product strength was lower than that of CTP-based formed coke.

#### 3.2.3. Consolidation System of Briquette Using Composite Binder as Binder

##### Effects of Phenolic Resin Ratio in the Composite Binder

**Figure 8:**
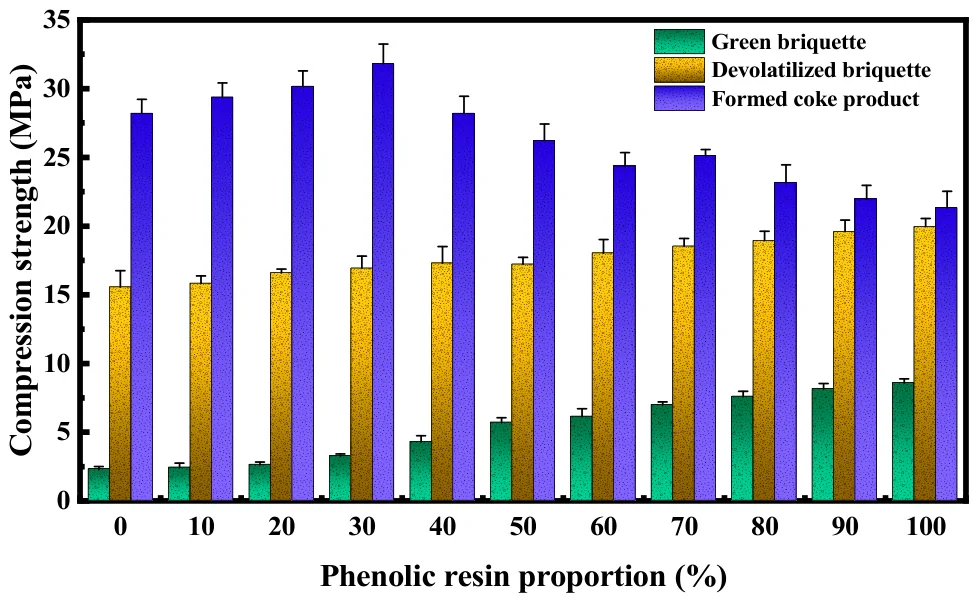
The effect of the phenolic resin ratio in the composite binder on the compressive strength of briquettes. Composite binder dosage: 12%; Briquetting pressure: 40 MPa. Devolatilizing condition: T = 370 °C, Time = 25 min. Carbonizing condition: T = 700 °C, Time = 30 min.

To harness the superior binding performance of PR at room temperature and the enhanced carbonization consolidation capability of CTP at elevated temperatures, a composite binder system was formulated in this study to leverage the complementary advantages of both binders. The objective was to produce formed coke with improved mechanical and thermal properties. The influence of the PR ratio in the composite binder on the compressive strength of briquettes was investigated, as shown in Figure 8.

The compressive strengths of both the green and devolatilized briquettes increased consistently with higher PR content. Their compressive strength increases from 2.30 MPa and 15.55 MPa to 8.57 MPa and 19.96 MPa, respectively, as the PR ratio increases from 0 to 100%. This trend is attributed to the superior binding and curing characteristics of PR, which enhance interparticle adhesion and structural integrity during the initial briquetting and devolatilization stages. In contrast, the strength evolution of the final formed coke products exhibited a more complex, non-monotonic dependence on the binder composition. As the PR ratio increased from 0% to 30%, the compressive strength of the formed coke rose from 28.21 MPa to a maximum of 31.82 MPa. This initial improvement suggests that a moderate addition of PR as an auxiliary binder strengthens the green and devolatilized briquettes, providing a more robust structural framework that facilitates the subsequent carbonization and consolidation driven primarily by CTP. However, further increasing the PR ratio beyond 30% led to a significant decline in the strength of the final product. This indicates that excessive PR content reduces the proportion of CTP, which is the primary contributor to high-temperature carbonization and consolidation. Consequently, the upper limit of the final product’s strength is constrained by an insufficient CTP dosage. Therefore, the optimal composition for the composite binder was determined to be 70% CTP and 30% PR, effectively balancing the complementary roles of the two binders to maximize the strength of the formed coke.

##### Effects of Composite Binder Dosage and Briquetting Pressure

**Figure 9:**
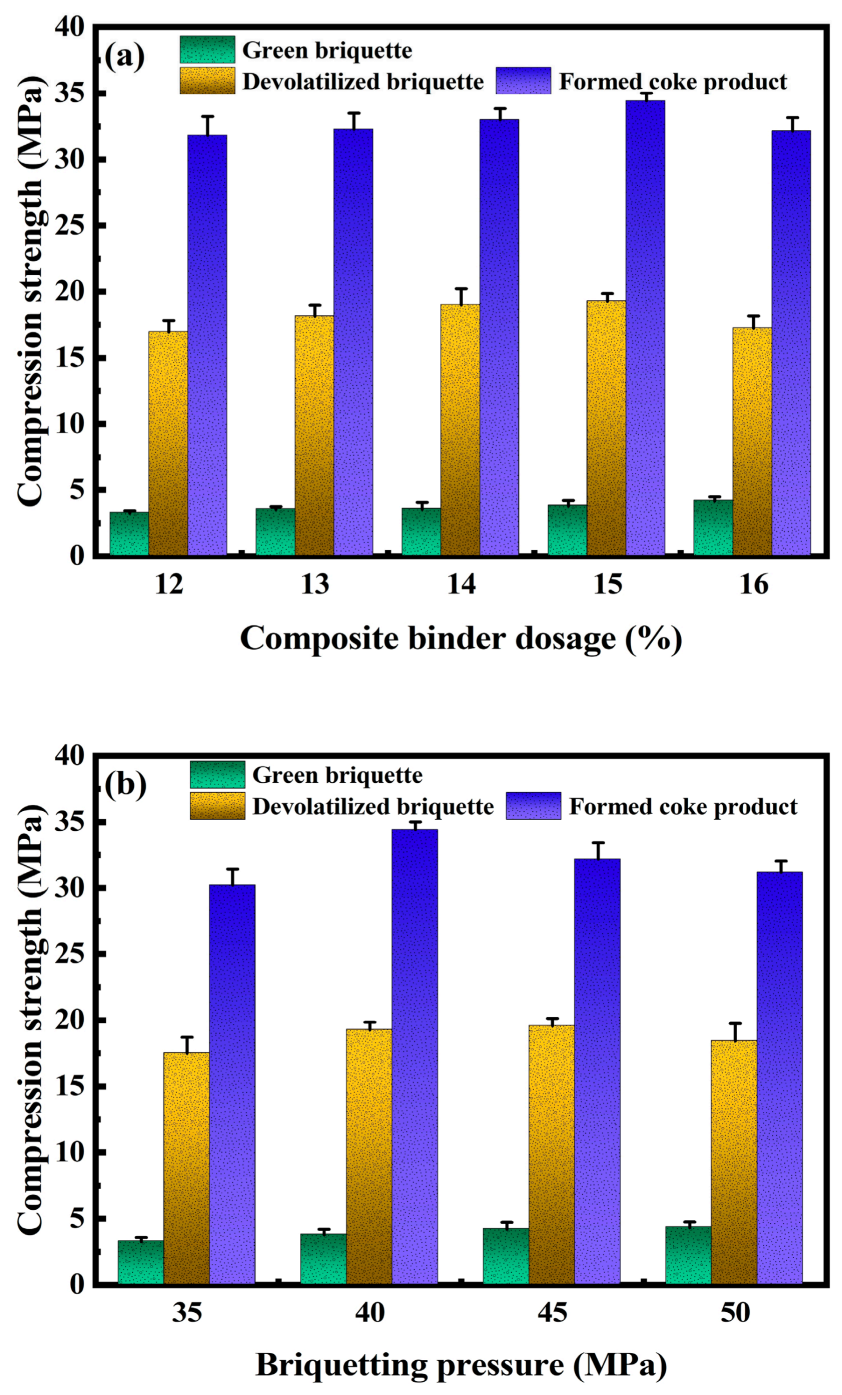
The effects of composite binder dosage (**a**) and briquetting pressure (**b**) on the compressive strengths of briquettes. Devolatilizing condition: T = 370 °C, Time = 25 min. Carbonization condition: T = 700 °C, Time = 30 min.

Following the determination of the optimal composition ratio for the composite binder, the influence of its dosage on the consolidation strength of the formed coke product was further investigated to identify the optimal binder dosage. The results are presented in Figure 9a.

The variation in compressive strength with composite binder dosage exhibited a trend similar to that observed for CTP alone, which is attributable to the dominant role of CTP as the primary binding component in the composite system. As the composite binder dosage increased, the compressive strengths of both the devolatilized briquettes and the final formed coke products rose, reaching maximum values of 19.25 MPa and 34.42 MPa, respectively, at a dosage of 15%. However, further increasing the binder dosage beyond this point resulted in a decline in strength, indicating that the optimal composite binder dosage is 15%. This optimum formulation corresponds to 10.5% CTP and 4.5% PR, which, while representing a higher total binder loading than the individual optima, reduces the usage of each individual binder (10.5 wt.% CTP and 4.5 wt.% PR, compared to 12 wt.% and 13 wt.%, respectively), achieving superior performance through the complementary action of the two components. Moreover, the resulting formed coke exhibited higher mechanical strength, demonstrating a complementary effect between the two binders. The effect of briquetting pressure on the compressive strength of briquettes prepared with the composite binder is illustrated in Figure 9b. The optimal briquetting pressure was determined to be 40 MPa, yielding a formed coke product with a compressive strength of 34.42 MPa. The high proportion of CTP in the composite binder results in a briquetting behavior that closely resembles that of CTP-based systems, thereby dictating the optimal pressure condition.

##### Effects of Devolatilizing and Carbonizing Conditions

**Figure 10:**
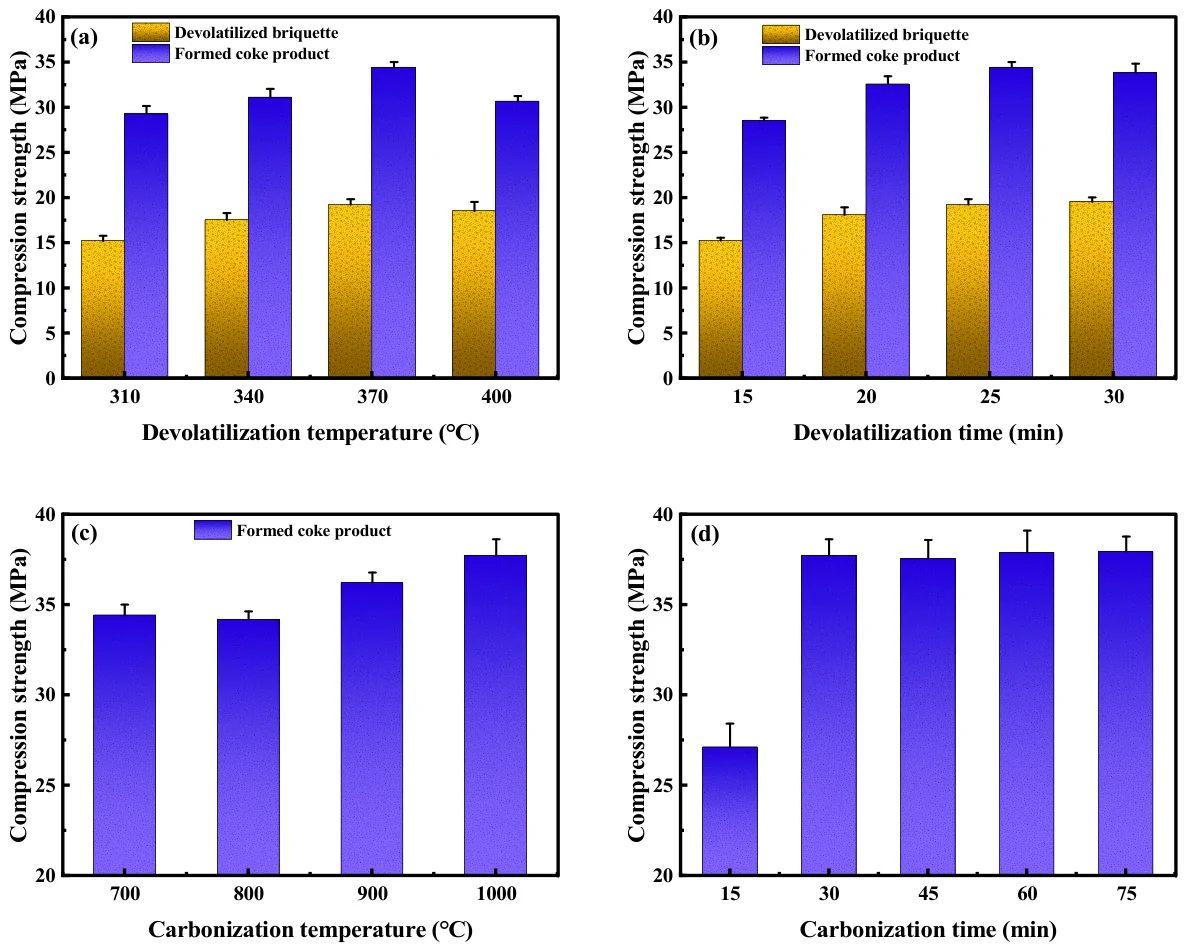
The effects of devolatilization temperature (**a**) and time (**b**), and carbonization temperature (**c**) and time (**d**) on the compressive strengths of briquettes. Composite binder dosage: 15%; Briquetting pressure: 40 MPa.

The influence of devolatilization and carbonization conditions on the mechanical strength of briquettes prepared with the composite binder was systematically examined, as shown in Figure 10.

As illustrated in Figure 10a,b, the effects of devolatilization temperature and time on the compressive strength of briquettes prepared with the composite binder exhibited trends similar to those observed for the single CTP binder. Moderately increasing the devolatilization temperature and extending the devolatilization time effectively enhanced the curing of the briquettes, providing a more robust structural foundation for subsequent high-temperature carbonization and consolidation. However, excessively high devolatilization temperatures led to an overly intense pyrolysis and volatilization of binder components within the briquette, thereby compromising its internal structural integrity and ultimately reducing the strength of the final formed coke product. The optimal devolatilization conditions were determined to be 370 °C for 25 min, yielding a formed coke with a compressive strength of 34.42 MPa. These devolatilization conditions were consistent with those identified for the CTP-only binder system, reflecting the dominant role of CTP within the composite binder during the devolatilization stage.

Similarly, as shown in Figure 10c,d, the influence of carbonization temperature and time on the strength of the formed coke prepared with the composite binder closely resembled the behavior observed for the single CTP system. Increasing the carbonization temperature effectively improved the compressive strength of the formed coke product, underscoring the superior carbonization and consolidation performance imparted by the CTP component within the composite binder. The strength of the formed coke reached 37.73 MPa at a carbonization temperature of 1000 °C. This value is substantially higher than the 32.92 MPa achieved with the single CTP binder under identical carbonization conditions. The enhanced performance can be attributed to the complementary role of PR in the composite system. PR contributes excellent binding and curing properties during the briquetting and devolatilization stages, producing devolatilized briquettes with improved structural integrity. This robust intermediate structure more effectively facilitates the subsequent carbonization and consolidation driven by CTP, ultimately leading to a stronger final product.

### 3.3. Strengthening Mechanism of Phenolic Resin on Carbonization Consolidation of Coal Tar Pitch

#### 3.3.1. Microstructure of Formed Coke Products

**Figure 11:**
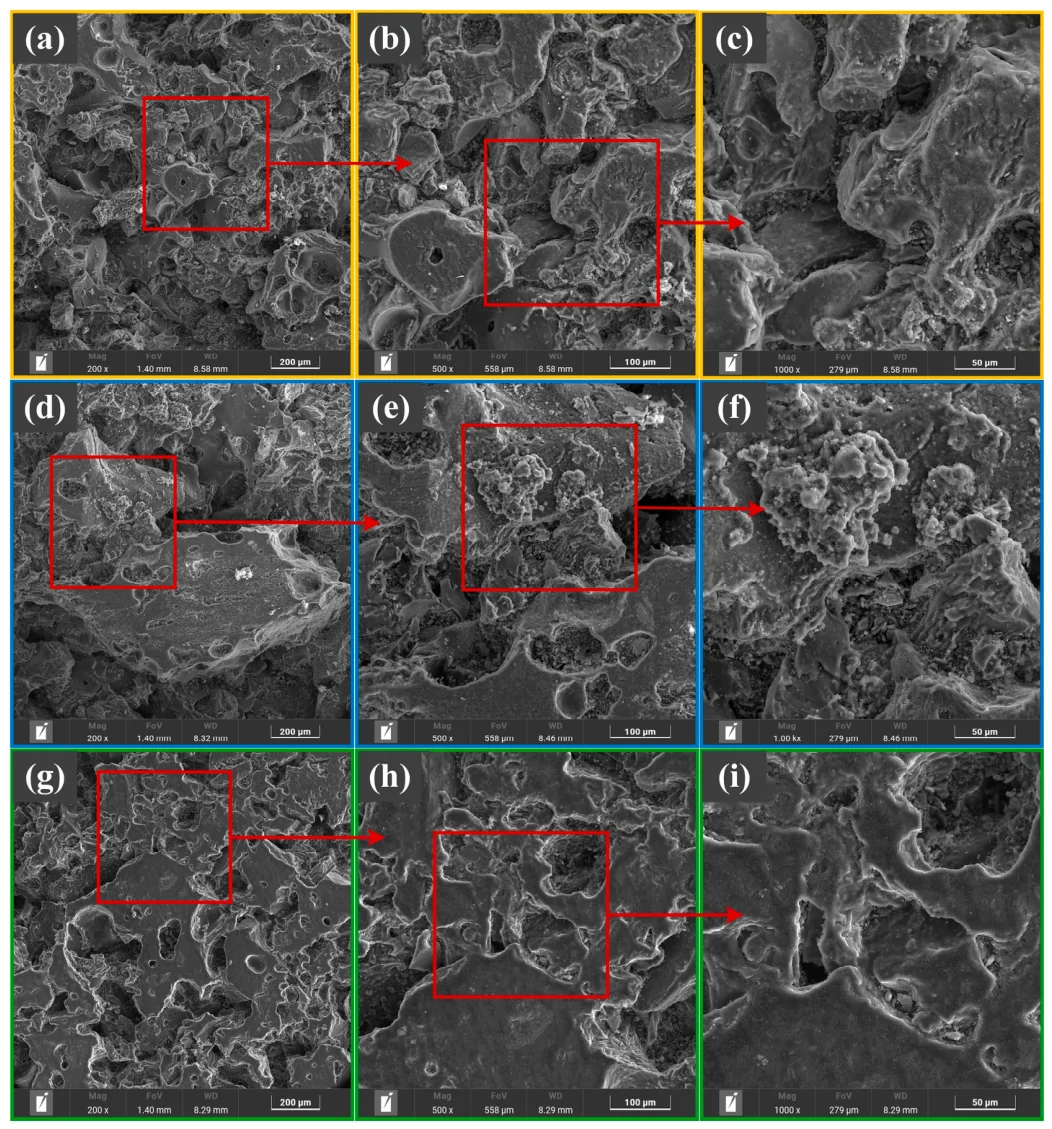
Microstructure of formed coke products obtained by using coal tar pitch (**a**–**c**), phenolic resin (**d**–**f**) and the composite binder (**g**–**i**) as the binder at the optimal conditions. Carbonization condition: T = 1000 °C, Time = 30 min.

To further elucidate the differences in internal adhesion and carbonization consolidation among CTP, PR, and their composite binder, the microstructure of ground and polished formed coke products was examined using scanning electron microscopy, as shown in Figure 11. In the formed coke product produced with PR as the sole binder (Figure 11d–f), substantial amounts of uncarbonized PR residues were observed, which formed large pores and cracks within the structure. These defects significantly compromise the mechanical and thermal strength of the product. In contrast, the internal particle bonding in the formed coke prepared with CTP (Figure 11a–c) was markedly improved, owing to the superior carbonization behavior of CTP. The carbonized consolidation formed by CTP effectively connects the coke breeze particles, creating a more integrated structure. However, the presence of numerous fine particles within the product indicates that although the strength was considerably enhanced compared to the PR-based product, it still does not meet the stringent requirements.

The microstructure of the formed coke produced with the composite binder (Figure 11g–i) demonstrated further enhancement. The addition of a small amount of PR effectively reinforced the carbonization consolidation driven by CTP. The PR filled the interparticle spaces within the briquette, and its strong binding and curing capabilities improved the adhesion state of CTP particles. This synergistic interaction further activated the carbonization and consolidation performance of CTP, resulting in a more robust carbonized network between the coke breeze particles. Consequently, the particles within the product were more tightly bonded, and the overall structure became more compact and continuous. This microstructural improvement explains the superior performance of the composite-binder-derived formed coke compared to those prepared with CTP or PR alone.

#### 3.3.2. Carbonization-Product Structure Defect Degree of Binders

**Figure 12:**
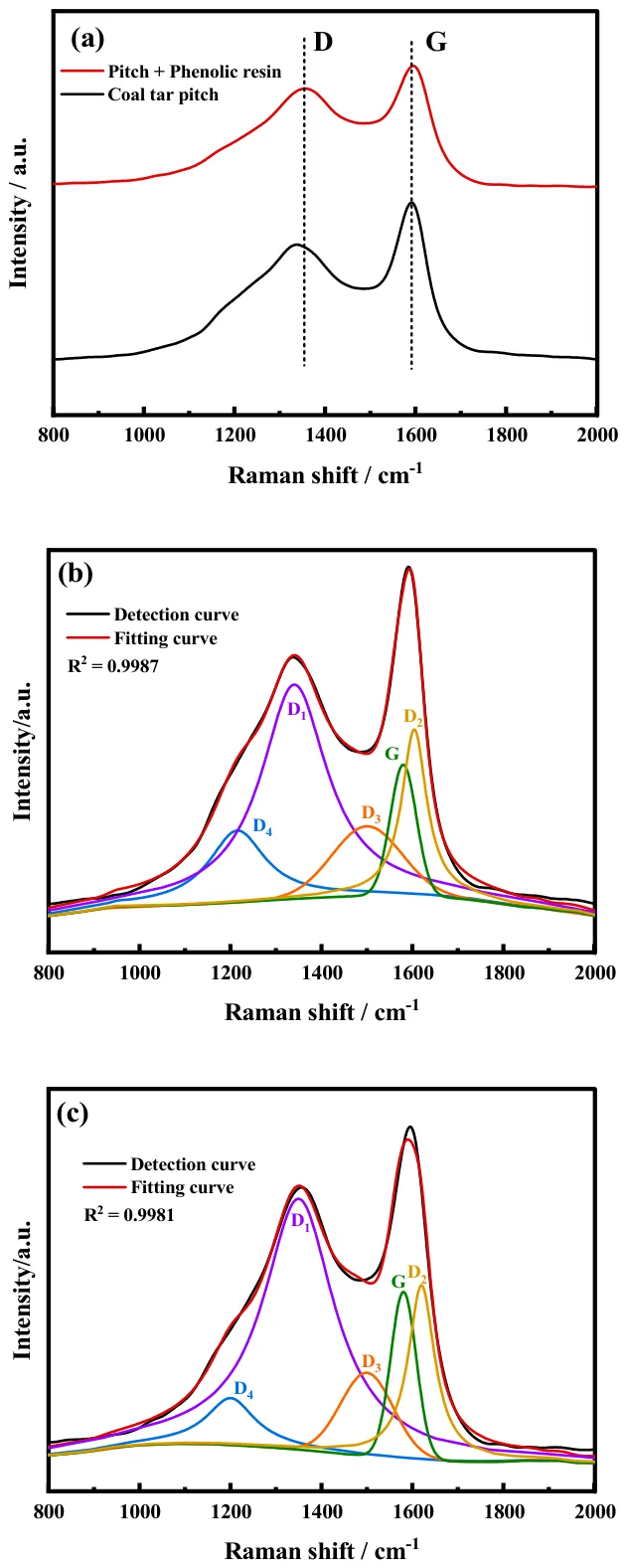
Raman spectra and peak fitting diagram of carbonization products obtained by coal tar pitch and composite binder. (**a**): Original Raman spectra; (**b**): peak fitting diagram of carbonization products from coal tar pitch at 1000 °C; (**c**): peak fitting diagram of carbonization products from composite binder at 1000 °C.

During the high-temperature carbonization process of formed coke, the carbonaceous organic compounds in the binder undergo carbonization via dehydrocondensation reactions, forming dense graphite-like aromatic lamellae. As carbonization proceeds, these aromatic lamellae approach each other through van der Waals forces and π-π stacking interactions, gradually stacking and consolidating to eventually form a continuous and dense carbonaceous network [41]. This carbon network acts as “bridging connections” distributed among the coke breeze particles within the formed coke [9]. Its structural integrity and crystalline ordering are key factors determining the overall mechanical properties and high-temperature stability of the formed coke. Therefore, this study conducted carbonization consolidation experiments on CTP and the composite binder, and employed Raman spectroscopy to analyze the structural integrity and defect degree of the final carbonized products, as shown in Figure 12.

To more precisely resolve the types and relative content of microstructural defects in carbonaceous materials, Raman spectroscopy typically deconvolves the D band (1350 cm^−1^, representing structural disorder in carbon materials) and the G band (1580 cm^−1^, corresponding to the E_2_g vibration mode of an ideal graphite lattice) into sub-peaks through curve fitting. The D band was further decomposed into four sub-peaks (D1–D4), each corresponding to structurally distinct defect origins, while the G band characterizes the intact graphite lattice vibration [42,43,44]. By fitting the raw spectra in Figure 12, the Raman deconvolution parameters for the carbonized products of CTP and the composite binder were obtained, as summarized in Table 3. The data show that the relative intensities of the D1 band (56.62%) and the G band (10.00%) in the carbonized composite binder are significantly higher than those in the CTP-only system (D1: 49.44%, G: 8.73%). This indicates more complete carbonization and consolidation in the composite binder system, where a greater number of graphite-like aromatic lamellae are formed and stacked, thereby yielding a stronger graphite crystal characteristic signal. Simultaneously, the increase in the number of aromatic lamellae correspondingly leads to more defect sites being detected, manifested as the relative enhancement of the D1 band. Moreover, concerning the D3 band (1500 cm^−1^, commonly attributed to amorphous carbon) and the D4 band (1200 cm^−1^, often associated with ionic impurities or disordered graphite lattices), the intensities in the carbonized composite binder are markedly lower than those in the CTP. This demonstrates that the incorporation of PR effectively reduces these two types of structural defects. The reasons are twofold: on one hand, the aromatic compounds in PR possess a higher initial condensation degree and greater chemical stability, resulting in a lower likelihood of cracking to form amorphous carbon at the carbonization temperature of 1000 °C, which correspondingly reduces the D3 band intensity; on the other hand, the ash content of PR is lower than that of CTP, thereby decreasing structural defects introduced by ionic impurities in the final carbonizate, as reflected by the lowered D4 band intensity.

In summary, the addition of a small amount of PR to CTP can significantly enhance the structural integrity and ordering of the carbonized products from the binder, while reducing defects related to amorphous carbon and impurities. However, as evidenced in Section 3.2, PR is not suitable as the dominant component: its highly condensed aromatic structure exhibits limited reactivity within the carbonization temperature window of formed coke, hindering further condensation and carbonization and thereby restricting continued enhancement of product strength.

#### 3.3.3. Carbonization-Product Microcrystalline Parameters of Binders

The carbonization and consolidation of CTP involve both chemical and physical transformations. Raman spectroscopy primarily characterizes the defect degree of the carbonized structure formed through dehydrogenative condensation, representing the chemical aspect of the process. In contrast, the physical transformation mainly refers to the rearrangement and stacking of large aromatic lamellae post-condensation, resulting in the formation of an ordered carbon layer structure.

**Figure 13:**
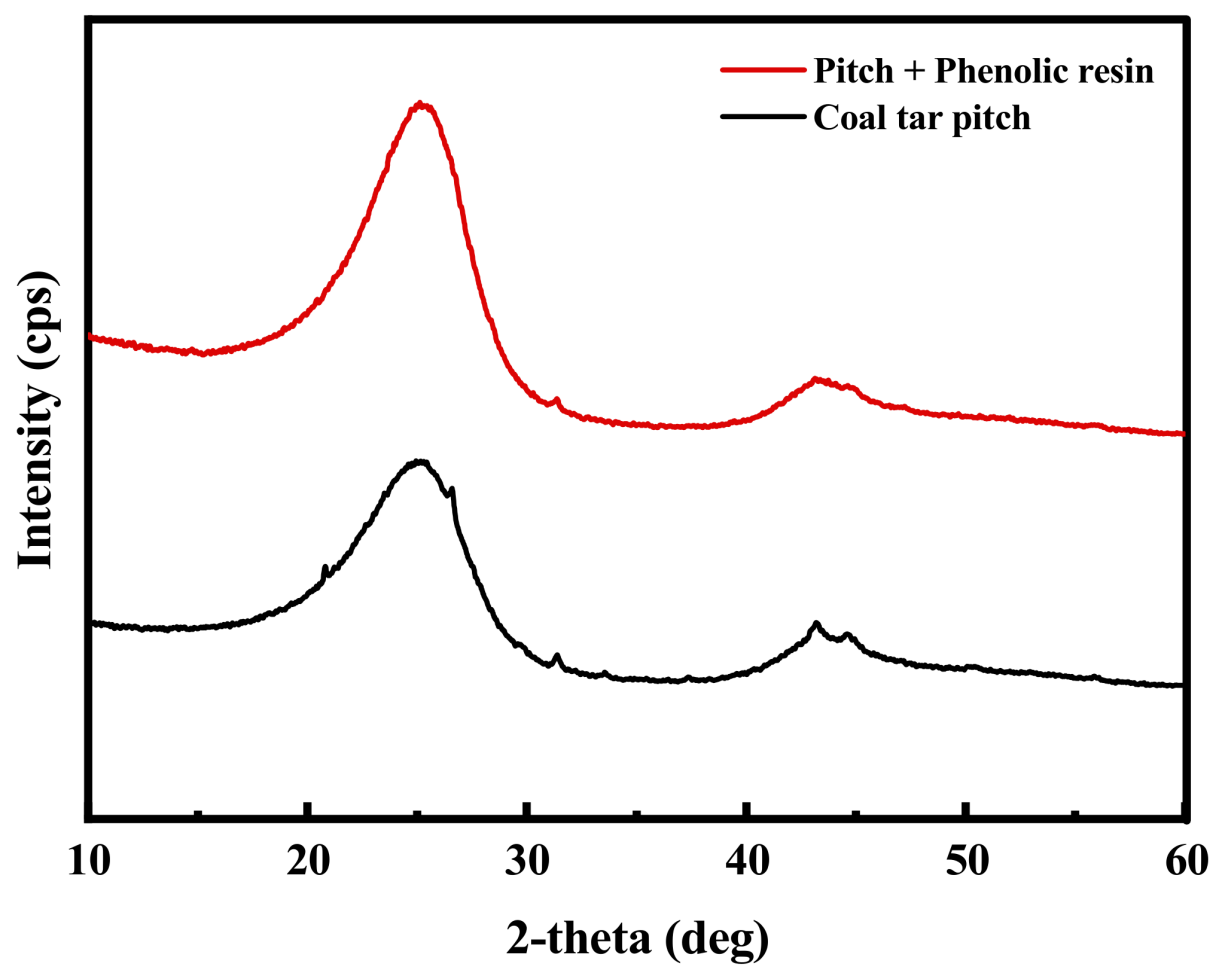
XRD patterns of carbonization products obtained by binders at 1000 °C.

To analyze the microstructure of the carbonized products from CTP and the composite binder and to calculate their microcrystalline stacking parameters, X-ray diffraction (XRD) analysis was performed. The results are presented in Figure 13. As shown, both carbonized products exhibit a distinct characteristic peak near 2θ ≈ 25°, indicating the presence of graphite-like microcrystals in each sample. The microcrystalline parameters derived from the XRD patterns are summarized in Table 4. The parameters include: d002, the interlayer spacing between aromatic sheets (nm); λ, the X-ray wavelength (0.15407 nm); θ002, the Bragg diffraction angle; LC, the average stacking height of aromatic sheets (nm); β002, the full width at half maximum (FWHM) of the diffraction peak; and N, the average number of stacked aromatic layers [45,46,47].

Compared to the carbonized product of CTP alone, the composite binder’s carbonized product exhibits a higher diffraction angle and a narrower FWHM. This indicates a stronger diffraction signal, a greater abundance of graphite-like microcrystals, and a more ordered turbostratic carbon. The calculated microstructural parameters further reveal that the carbonized product of the composite binder possesses a smaller interlayer spacing (d002), a greater stacking height (LC), and a higher number of stacked aromatic layers (N). These results demonstrate that the inclusion of phenolic resin promotes a more ordered and compact carbon structure with enhanced carbonization degree. This conclusion is consistent with the results in Section 3.2. The formation of such a highly carbonized and consolidated structure is conducive to establishing stronger “bridging connections” between coke breeze particles within the formed coke, thereby contributing to the improved mechanical strength of the final product.

### 3.4. Performance of Formed Coke Product

#### 3.4.1. Proximate Analysis, Ultimate Analysis and Calorific Values

The proximate and ultimate analyses of the formed coke products prepared using CTP, PR, and the composite binder under optimal conditions are presented in Table 5. The product prepared with PR as the binder (PR-FC) exhibited the highest fixed carbon content (87.53 wt.%) and the lowest volatile matter content (0.72 wt.%), consistent with the inherent high carbon content and low volatility of PR. In contrast, the product produced with CTP alone (CTP-FC) displayed a lower fixed carbon content (85.85 wt.%) and a higher volatile content (1.53 wt.%), reflecting the relatively higher volatile components in CTP. The product derived from the composite binder (CB-FC) exhibited intermediate values, with a fixed carbon content of 86.31 wt.% and a volatile matter content of 1.21 wt.%, aligning with the proportional blending of the two binder constituents. Similarly, the ultimate analysis and calorific values followed comparable trends, with CB-FC showing properties between those of CTP-FC and PR-FC.

#### 3.4.2. Coke Reactivity Index (CRI) and Coke Strength After Reaction (CSR)

**Figure 14:**
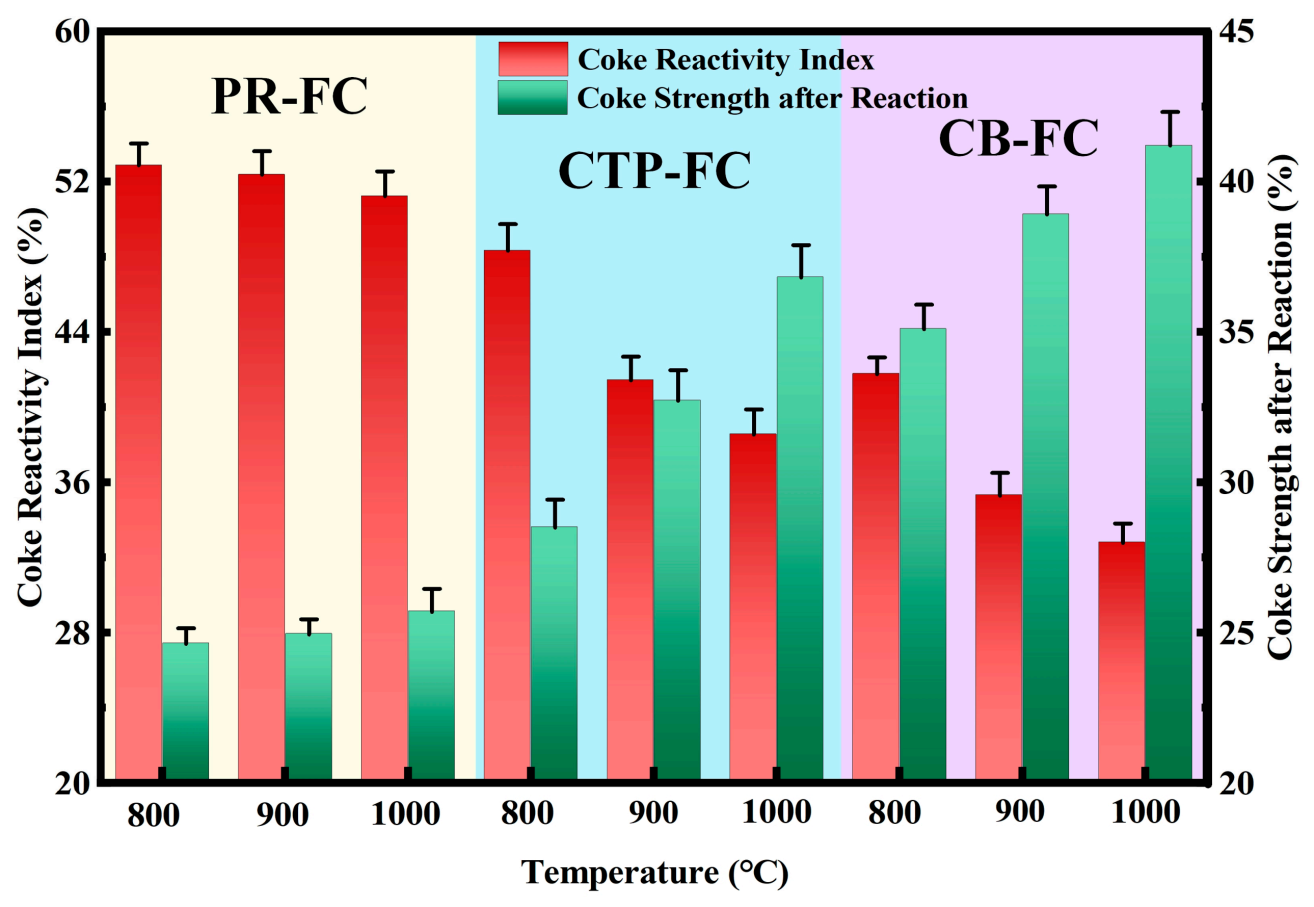
Coke reactivity index (CRI) and coke strength after reaction (CSR) of formed coke products.

The thermal stability and structural integrity of coke under high-temperature conditions are critical for its industrial application. These properties are typically assessed using two standardized metrics: the Coke Reactivity Index (CRI) and the Coke Strength after Reaction (CSR). In this study, the CRI and CSR of formed coke products prepared from PR, CTP, and the composite binder at carbonization temperatures of 800 °C, 900 °C, and 1000 °C were systematically evaluated, as illustrated in Figure 14.

The results indicate that the formed coke produced with the composite binder exhibited superior thermal strength compared to those prepared with either CTP or PR alone. This enhancement aligns with the compressive strength trends presented in Section 3.2, confirming that the synergistic interaction between PR and CTP within the composite binder contributes to a more coherent and robust internal structure. Specifically, the composite binder leverages the excellent binding properties of PR at lower temperatures and the superior carbonization consolidation capability of CTP at elevated temperatures, resulting in a product with improved resistance to thermal degradation. Furthermore, increasing the carbonization temperature significantly improved the thermal performance of the composite-binder-derived formed coke. At 1000 °C, the product demonstrated a CRI of 32.82% and a CSR of 41.21%, representing a notable enhancement in thermal stability. These values suggest that the formed coke possesses adequate mechanical and thermal integrity for potential applications. It could serve as a reducing agent in non-ferrous metallurgical processes (e.g., copper or lead smelting) or as a partial substitute for high-quality metallurgical coke in ironmaking blast furnaces, where thermal endurance is essential [48,49].

## 4. Conclusions

This study successfully developed and validated a composite binder system, integrating phenolic resin (PR) and coal tar pitch (CTP), for producing high-strength metallurgical coke substitutes from coke breeze. The composite design capitalizes on the complementary properties of the two binders: PR exhibits excellent binding and curing capabilities, enabling the formation of high-strength green and devolatilized briquettes, yet demonstrates inferior carbonization consolidation at elevated temperatures. Conversely, CTP shows relatively weak room-temperature adhesion but offers superior high-temperature carbonization behavior. By combining them, the system leverages PR’s strong initial binding and curing to establish a robust structural framework, which is subsequently reinforced and consolidated by the effective carbonization of CTP during high-temperature treatment. The optimal composite binder formulation was determined to be a CTP-to-PR mass ratio of 7:3, with a dosage of 15 wt.% relative to coke breeze. The recommended processing conditions included a briquetting pressure of 40 MPa, devolatilization at 370 °C for 25 min, and carbonization at 1000 °C for 30 min. This integrated approach yielded a formed coke product with a compressive strength of 37.73 MPa and a Coke Strength after Reaction (CSR) of 41.21%, significantly outperforming products prepared with individual binders. Microstructural analysis confirmed a more compact and continuous carbon network, supporting the enhanced mechanical and thermal stability observed. Furthermore, the analysis of carbonized binder structure shows that the addition of a small amount of PR to CTP can significantly enhance the structural integrity and ordering of the carbonized products from the binder, while reducing defects related to amorphous carbon and impurities. This work presents a promising and sustainable strategy for coke breeze valorization, producing a viable substitute with potential applications in metallurgical processes, thereby contributing to resource conservation and environmental sustainability. The findings provide a transferable framework for the recycling of solid fuel waste, offering new insights into waste-to-fuel conversion.

## Figures and Tables

**Table 1:** Proximate analysis and particle size composition of coke breeze. (wt.%).

Proximate Analysis ± SD (Wet Base)				Particle Size Composition/mm			
Fixed carbon	Volatile matter	Ash	Moisture	>3	1–3	0.5–1	≤0.5
84.88 ± 0.055	1.41 ± 0.012	12.12 ± 0.027	1.59 ± 0.016	5.55	8.25	22.14	64.06

**Table 2:** Proximate analysis, ultimate analysis and softening temperature of coal tar pitch (wet base).

Samples	Proximate Analysis ± SD (wt.%)				Ultimate Analysis (wt.%)				Softening Temperature (°C)
	Fixed Carbon	Volatile Matter	Ash	Moisture	C	H	O	N	
CTP	38.81 ± 0.032	60.02 ± 0.048	1.11 ± 0.015	0.06 ± 0.001	89.51	3.64	3.09	0.67	85
PR	58.03 ± 0.049	41.21 ± 0.048	0.68 ± 0.0005	0.08 ± 0.0005	91.52	2.08	5.11	0.95	-

**Table 3:** Raman spectrum fitting parameters of carbonization products obtained by coal tar pitch and composite binder.

Binder		Peak				
		D1	D2	D3	D4	G
Coal tar pitch	Area (a.u.)	1,703,530	526,637	423,191	491,400	300,808
	Ratio (%)	49.44	15.28	12.28	14.26	8.73
Composite binder	Area (a.u.)	1,706,765	476,807	307,598	221,740	301,590
	Ratio (%)	56.62	15.82	10.20	7.36	10.00

**Table 4:** Microcrystalline structure parameters of carbonization products obtained by coal tar pitch and composite binder at 1000 °C.

Binder	2θ (rad)	B (rad)	d002 (nm)	Lc (nm)	N
Coal tar pitch	24.79	4.885	0.359	1.647	5.59
Composite binder	25.23	4.517	0.353	1.783	6.05

**Table 5:** Proximate analysis, ultimate analysis and calorific values of formed coke products (dry basis).

Samples	Proximate Analysis (wt.%)			Ultimate Analysis (wt.%)				Calorific Values (Mj/Kg)
	FC	VM	Ash	C	H	N	O	
CTP-FC	85.85	1.53	12.55	82.52	1.18	0.66	2.81	31.05
PR-FC	87.53	0.72	11.75	82.98	0.49	0.53	2.54	30.78
CB-FC	86.31	1.21	12.48	82.58	0.92	0.62	2.69	30.97

## Data Availability

The original contributions presented in this study are included in the article. Further inquiries can be directed to the corresponding authors.
